# Pharmacists and telemedicine: an innovative model fulfilling Sustainable Development Goals (SDGs)

**DOI:** 10.1186/s40545-021-00378-9

**Published:** 2021-11-08

**Authors:** Nadia Bukhari, Maryam Siddique, Nazia Bilal, Sobia Javed, Arzu Moosvi, Zaheer-Ud-Din Babar

**Affiliations:** 1 UCL School of Pharmacy, 29-39 Brunswick Square, London, WC1N 1AX UK; 2 doctHERs, Karachi, Pakistan; 3 Department of Pharmacy, University of Huddersfield, Queensgate, Huddersfield, HD1 3DH UK

**Keywords:** Pharmacists, Telemedicine, Sustainable Development Goals

## Abstract

The lack of access to safe medicines and quality healthcare services in peri-urban and rural areas is a major challenge driving a health system to innovate new models of care. This commentary will discuss the implementation and impact of the “Guddi baji” tele-pharmacy model, a project piloted by doctHERs, one of Pakistan’s leading telemedicine organizations. This innovative model has described the reintegration of women into the workforce by leveraging technology to improve the level of primary health care services and contributes to safe medication practice in a remote area. Our intervention proposed the deployment of technology-enabled, female frontline health workers known as the Guddi baji (meaning The Good Sister) in a rural village. They serve as an “access point to health care” that is linked to a remotely located health care professional; a licensed doctor or a pharmacist within this model.

## Introduction

Pakistan has a population of over 220 million [1], however there are challenges within its healthcare sector. These include limited accessibility to reliable healthcare services, access to authenticated medicines, and limited transportation to essential healthcare facilities. Challenges specifically due to the COVID-19 pandemic [2] include accessibility to quality healthcare services due to social distancing, limited capacities within healthcare facilities, and mental health limitations such as social anxiety. With these challenges, countries such as Pakistan have shown great need and emphasis for ease of accessibility to healthcare at the comfort of their home [3].

Strategically, healthcare professionals can help to decrease the challenges in Pakistan by creating a platform where access to doctors, pharmacists, and other relevant healthcare professionals is technology based, easy to navigate, and at the comfort of one’s home. Telemedicine has become an on-going development within the country, increasing the importance of patient access to quality healthcare services, which in turn increases literacy on individualized health [4]. Pharmacists in particular can revolutionize the progress of digital health into multiple directions to bypass the challenges that hinders these interventions to improve the health care system in Pakistan [5].

doctHERs [6] is a gender inclusive social enterprise which is unique in that it uses a digital health platform to match the underutilized capacity of female health care professionals like doctors, pharmacists, nurses, etc. (who would otherwise be excluded from the workforce) to the unmet needs of health seekers in emerging markets. It capacitates, and equips previously under-employed, female frontline health workers with laptops, tablets, smartphones, with 4G Wi-Fi devices.

At doctHERs [6], a number of pharmacists are involved in the expansion of telemedicine services for inception and development of pharmacy services through a pilot project. Pharmacists are considered medication experts within the healthcare field. One of the key roles and services of the pharmacist as a qualified health care professional includes medication counselling and chronic disease management which enables good pharmacy practice [7]. These services can easily be performed remotely through a telemedicine platform. Through e-consultations, pharmacists are remotely connected with patients and a network of healthcare professionals to provide medication management services ensuring the safe use of medicines via patient counselling.

This commentary describes the expansion of pharmacy services; by leveraging technology and giving access to over-the-counter medicines to the rural community at the ‘at-point-of-need’. Furthermore, this commentary describes the model, implementation and impact of an innovative rural e-pharmacy model in Pakistan.

### The project fulfilling Sustainable Development Goals (SDGs)

Over 80% of rural communities in Pakistan lack access to affordable, quality healthcare contributing to the high rates of infant and maternal mortality in South Asia. The debilitating effects of illness and disease lead to loss of productivity, social isolation and/or exclusion and a downward socioeconomic spiral for rural, hard-to-reach families which keeps them trapped in poverty due to a gap in health allocations and expenditure by government [8, 9].

Major contributing factors to the lack of access to quality healthcare, Sustainable Development Goal 3 (SDG3) [10], in rural areas include:

(i) Lack of skilled, technology-enabled frontline health workers in rural village communities.
(ii) 50% of the medicines dispensed and sold from urban factories to retail pharmacies are reportedly counterfeit or spurious [11].
(iii) Exclusion of qualified female health care professionals in the workforce in both developing countries and mature markets has resulted in not only a massive loss of human potential but also loss of human life—especially in countries such as Pakistan, where millions of people continue to lack access to quality, affordable healthcare. Most of these female medical graduates are not participating in the workforce—largely due to sociocultural barriers that prevent them from achieving their professional aspirations with their family responsibilities [12].

Therefore, doctHERs [6] have been reintegrating these female health care professionals into the health workforce by supporting gender equality, Sustainable Development Goal 5 (SDG5) [8], and creation of decent work and economic growth opportunities, Sustainable Development Goal 8, (SDG8) [10].

### The* Guddi Baji *Program: a multistakeholder collaboration for tele-pharmacy model

In collaboration with TRANSFORM [13] and Unilever Pakistan, doctHERs [6] launched a sustainable development initiative to reach 3 million in rural Pakistan who lack access to affordable, quality healthcare. TRANSFORM is a joint initiative between Unilever and the UK’s Foreign, Commonwealth & Development Office (FCDO) and EY to support business models and behavior change interventions to deliver market-based solutions for low-income household needs.

The pilot project was known as the *Guddi Baji Program* [14] which is aimed to leverage the widespread reach of Unilever’s rural retail program to deliver high-quality health and wellness services for 1 million women across 3000 villages in Pakistan. In this project, doctHERs upskilled, equipped and deployed 100 technology-enabled Guddi Bajis (GB) (*Guddi Baji* meaning The Good Sister). These Guddi Bajis are frontline female health workers in rural villages across three provinces of Pakistan; Sindh, Punjab and Khyber Pakhtunkhwa. These health workers provide awareness on various women’s health issues and connect female patients to a nationwide network of female doctors via high definition (HD) video consultation.

**Fig. 1:**
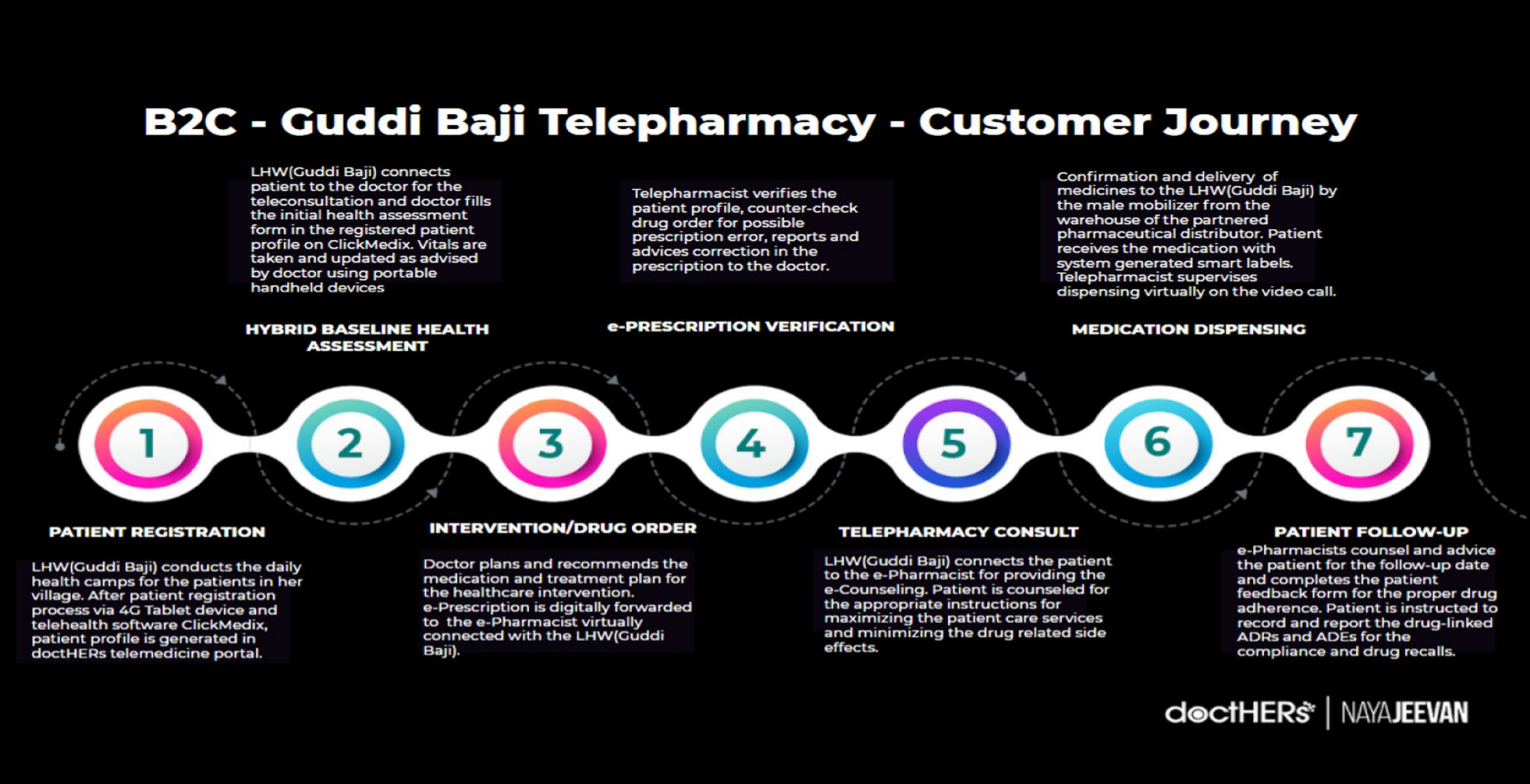
Guddi Baji (GB) tele-pharmacy model

Project TRANSFORM was upscaled by team doctHERs for a successful tele-pharmacy intervention in the underserved communities. After introducing the model with a pilot district, positive impacts are reached as locals are getting the last-mile access to quality healthcare by bringing transforming changes in practices like halting the use of substandard and falsified medicines ensuring safe medication practice (Fig. 1).

The tele-pharmacy pilot was launched in December 2020 with 31 GBs and 4 female pharmacists of the Bahawalpur district. GBs under this initiative sell over-the-counter (OTC) medicines door to door under the supervision of a registered female pharmacist. These medicines were carefully selected by the Chief Pharmacist and the e-Pharmacy Manager taking into consideration the risks and benefits of the OTC medicines as well as meeting the needs of the local population (see Table 1).

**Table 1:** Generic medicines and indication

Generic medicines	Indication
Paracetamol	Pain, fever
Orphenadrine + paracetamol	Generalized pain
Iodine/methylsalicylate	Body pain, generalized pain (topical)
Calcium carbonate/magnesium carbonate	Dyspepsia
D-Pantothenol	Wounds, infections (anti-septic)
Cetirizine dihydrochloride	Allergy
Ivy leaf extract	Cough, sore throat
Electrolytes	Electrolyte deficiency
Vitamins + calcium + minerals	Supplements
Iron supplement + folic acid	Supplements

The primary objective for the pilot was to add generic medicines from the categories indicated for management of pain, fever, flu, allergies, respiratory distress, cough, heartburn, topical infections, iron deficiency, vitamin deficiency, folic acid deficiency and electrolyte imbalance. The medicines were selected by taking the following into consideration: (a) disease prevalence in the community; (b) category/drug of choice in common health issues; (c) brands with good market ranking; (d) preferred SKUs and dosage forms, and (e) good-quality packaging with nominal MRPs.

**Fig. 2:**
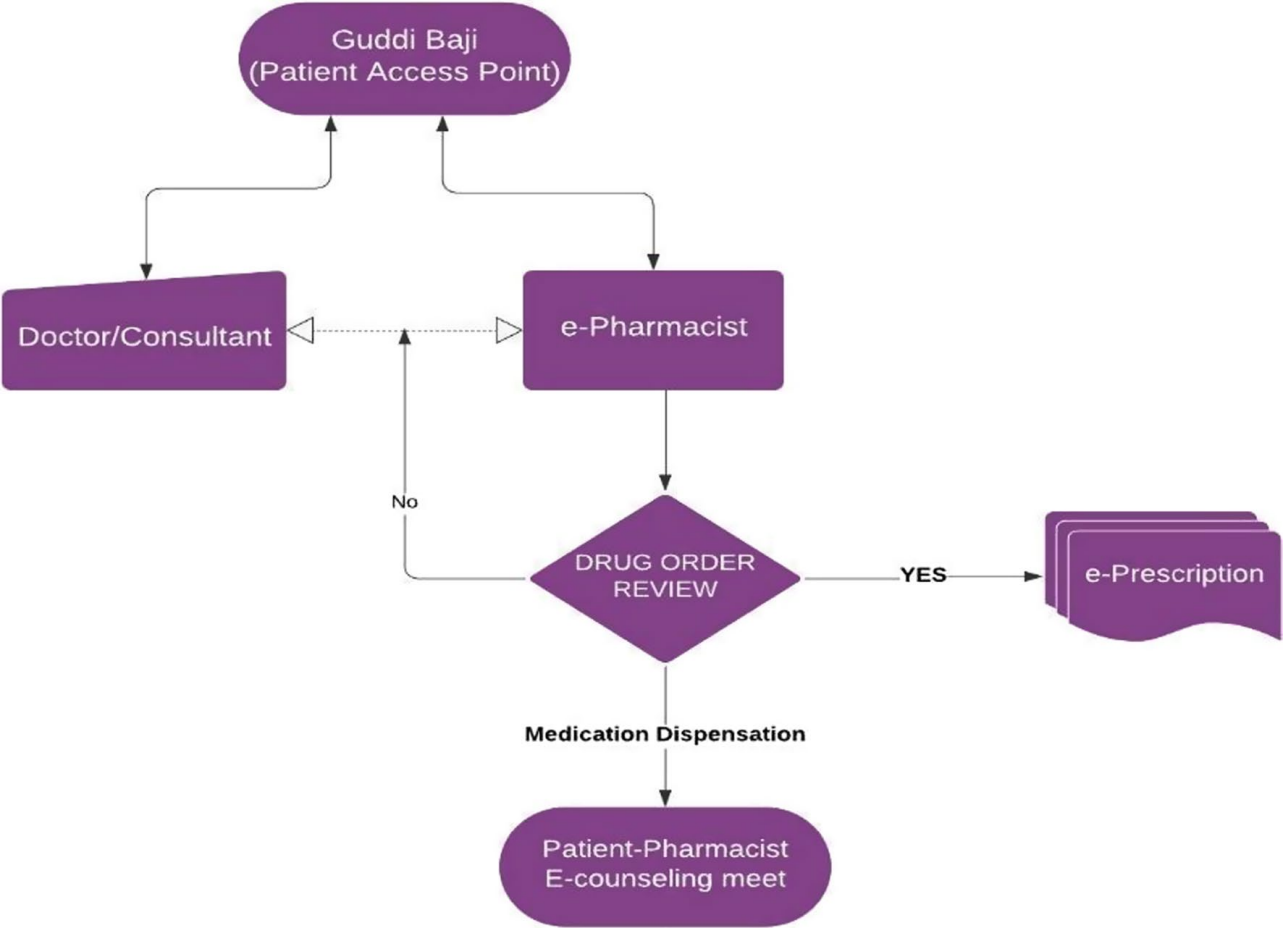
Process flow for tele-pharmacy intervention

During her door-to-door visits, the GB connects to either the doctor or the pharmacist before selling the OTC medicine to the patient. If the GB has connected to the doctor first, she is still required to connect to the pharmacist who authorizes the sale of the OTC medicine and provides the necessary counselling and follow up with the patient.The GBs have been specifically trained by the chief pharmacist and e-pharmacy manager on the indications and supply and storage of the OTC medicines as well as understanding the role of the pharmacist. They have also been made fully aware that legislatively, no sale of medicines can be made without the authorization of the pharmacist, even if the OTC medicine has been recommended by the doctor (Fig. 2).

The sale of the OTC medicines is authorized by the pharmacist via digitally generated e-prescriptions. In addition, the medicines are delivered, and stored under the supervision of the pharmacist and the GBs are provided with light, moisture and temperature-sensitive bags to correctly store the medicines.

### Impact: post-execution of the tele-pharmacy pilot

After 3 months of successive implementation and on-going tele-pharmacy services integrated with the telemedicine and GB patient visits, the following changes were observed in the target district (Bahawalpur):

1. *Improvement in proactive health-seeking behavior* for the reduction of micronutrient deficiencies and iron/folate supplementation for pregnant women, smoking cessation, active lifestyle, nutritional supplementation (nutraceuticals) for improved maternal health and prevention of diseases.
2. *Involvement of licensed female pharmacists at time of medicine dispensation* end-consumers are valuing and availing the opportunity to receive instructions on dose administration and any potential side-effects from online, licensed, female pharmacists. Medicines are handled, delivered, and stored under the supervision of our qualified, registered pharmacists. Our e-pharmacists ensure proper dispensation of medicines after the e-counseling via digital e-pharmacy platform.
3. *Preventing the sale and consumption of counterfeit medications* Tele-pharmacy transform 2.0 focused on patients having immediate and affordable access to quality patient care, simultaneously, preventing the use of substandard, adulterated, and falsified medicines.
4. *Better patient compliance* daily follow-ups with patients by the Female Health Workers (FHWs)—scheduled home health visits and virtual counseling for medication dispensation and intake by licensed female pharmacists (who can also counsel patients on side effect management) has increased the patient satisfaction and compliance.
5. *Cost-effective accessibility *we have provided innovative cost-reduction measures by lowering the logistic/transportation-related opportunity costs that patient incur in commuting to basic health units and other facilities in the city (~ distance of 30–100 kms) for the purchase of medicines and other healthcare products.

## Conclusion

Delivering quality healthcare at the last-mile can be incredibly challenging in rural communities in developing countries, such as Pakistan. Via project TRANSFORM, doctHERs have impacted over 1 million lives within rural Pakistan [14].

This pilot program has significantly influenced patient adherence and health awareness within the rural communities. It has bridged the gaps between the patient and community health workers in a primary care setting by leveraging technology. The findings indicate that the re-integration of pharmacists into the health care delivery pathway in order to overcome primary health care issues in a rural community, is promising. This model has demonstrated the expansion of the role of the pharmacist by engaging them in a seamless virtually integrated e-health system. This in turn has generated patient health awareness and improved drug adherence by addressing the communication gaps within the primary care setting. This intervention has contributed to the prevention of medication errors as the model enables the rural community to access quality healthcare services as well as have access to a trained health care professional in real time, ensuring safe medication practices. Similar to this rural tele-pharmacy intervention, doctHERs aims to continue working towards the implementation of innovative, sustainable, evidence-based healthcare solutions.
